# Organelle Cooperation in Stem Cell Fate: Lysosomes as Emerging Regulators of Cell Identity

**DOI:** 10.3389/fcell.2020.00591

**Published:** 2020-07-07

**Authors:** Lisa M. Julian, William L. Stanford

**Affiliations:** ^1^Department of Biological Sciences, Simon Fraser University, Burnaby, BC, Canada; ^2^Regenerative Medicine Program, Ottawa Hospital Research Institute, Ottawa, ON, Canada; ^3^Department of Cellular and Molecular Medicine, University of Ottawa, Ottawa, ON, Canada; ^4^Ottawa Institute of Systems Biology, Faculty of Medicine, University of Ottawa, Ottawa, ON, Canada; ^5^Department of Biochemistry, Microbiology and Immunology, University of Ottawa, Ottawa, ON, Canada

**Keywords:** lysosomes, stem cell identity and fate, metabolism, neural stem cell (NSC), pluripotent stem cell (PSC), neural crest (NC), cancer stem cell (CSC)

## Abstract

Regulation of stem cell fate is best understood at the level of gene and protein regulatory networks, though it is now clear that multiple cellular organelles also have critical impacts. A growing appreciation for the functional interconnectedness of organelles suggests that an orchestration of integrated biological networks functions to drive stem cell fate decisions and regulate metabolism. Metabolic signaling itself has emerged as an integral regulator of cell fate including the determination of identity, activation state, survival, and differentiation potential of many developmental, adult, disease, and cancer-associated stem cell populations and their progeny. As the primary adenosine triphosphate-generating organelles, mitochondria are well-known regulators of stem cell fate decisions, yet it is now becoming apparent that additional organelles such as the lysosome are important players in mediating these dynamic decisions. In this review, we will focus on the emerging role of organelles, in particular lysosomes, in the reprogramming of both metabolic networks and stem cell fate decisions, especially those that impact the determination of cell identity. We will discuss the inter-organelle interactions, cell signaling pathways, and transcriptional regulatory mechanisms with which lysosomes engage and how these activities impact metabolic signaling. We will further review recent data that position lysosomes as critical regulators of cell identity determination programs and discuss the known or putative biological mechanisms. Finally, we will briefly highlight the potential impact of elucidating mechanisms by which lysosomes regulate stem cell identity on our understanding of disease pathogenesis, as well as the development of refined regenerative medicine, biomarker, and therapeutic strategies.

## Metabolic Organelle Networks Regulate Stem Cell Fate

Stem cell fate decisions, including the determination of cellular identity, are intimately linked to gene expression networks with each decision driven by wide-spread transcriptional changes (Walker et al., 2007; Julian et al., 2013, 2017b; Julian and Blais, 2015; Pinto et al., 2018; Abdolhosseini et al., 2019; Wells and Choi, 2019). Transcriptional regulation does not function in isolation, however. Cell fate decisions are heavily influenced by dynamic communication between the nucleus with multiple biological processes and signaling cascades involving macromolecule interactions at the cell membrane, throughout the cytoplasm, and within or on the surface of organelles (Shah et al., 1996; Zhou et al., 2009; Julian et al., 2013; Julian and Blais, 2015; Singh et al., 2015; Chen et al., 2016; Young et al., 2016; Khacho and Slack, 2017b; Chang et al., 2018; Obernier et al., 2018; Bahat and Gross, 2019; Jaiswal and Kimmel, 2019; Kinney et al., 2019; Shlyakhtina et al., 2019; Chang, 2020). The successful development, long-term homeostasis, and post-injury repair of organs and tissues is dependent on the pliability in cell fate decisions that these integrated biological networks permit. Yet, this flexibility can also give rise to the aberrant stem cell populations that underlie pathology in many diseases including tumor syndromes. This double-edged sword of cell fate pliancy is paralleled by dynamics in metabolic signaling pathways. It is therefore crucial to understand the breadth of regulatory processes and factors that contribute to the biological networks underlying metabolic control and to elucidate their impacts on cell fate.

### Metabolic Signaling Impacts Cell Identity

Cellular metabolism is broadly defined by the numerous biochemical pathways that participate in the processing of nutrients including amino acids, carbohydrates, and lipids, into their primary molecular building blocks along with energy in the form of adenosine triphosphate (ATP). The ATP produced through metabolic pathways is necessary to fuel the myriad of biological processes that take place within a cell to support its growth and viability. Metabolic bioenergetic signaling pathways are closely integrated with organelle-based processes, which together focus on balancing anabolic and catabolic cellular activities (Thelen and Zoncu, 2017; Todkar et al., 2017; Gordaliza-Alaguero et al., 2019; Xia et al., 2019). Respectively, anabolism and catabolism function to drive the production versus degradation of biomass, which is necessary to balance cell and tissue growth, homeostasis, and survival. Metabolic pathways are highly interconnected and dynamic, focused on managing supply and demand of available energy and nutrient substrates in the face of ever-changing environmental conditions. While many bioenergetic pathways exist that contribute to ATP production, a cell can be classified based on its relative reliance on two over-arching processes: oxidative phosphorylation (OxPhos) and glycolysis. These processes drive differential rates of ATP production and a unique complement of metabolite by-products (Folmes et al., 2012; Zhang et al., 2018; Gordaliza-Alaguero et al., 2019; Intlekofer and Finley, 2019). Oxidative phosphorylation is linked to the mitochondrial electron transport chain (ETC) and fueled by energy precursors generated through the tricarboxylic acid cycle (TCA). Alternatively, glycolysis takes place in the cytoplasm and can provide pyruvate as a substrate to feed into the TCA or can permit fully anaerobic macromolecule metabolism.

In addition to the production of ATP, metabolic signaling is now known to exert wide-ranging effects within a cell and has in fact emerged as a primary biological process underlying the regulation of cell fate decisions in a wide range of cell types, including stem cell populations. These impacts are achieved through mechanisms that include regulation of transcription factor expression and localization, integration of metabolic networks with growth factor and developmental signaling pathways, and the production of cell type and context-specific metabolites that permit chemical modification of signaling cascades and epigenetic regulation of DNA and histone proteins (Chung et al., 2007; Khacho et al., 2016, 2019; TeSlaa et al., 2016; Young et al., 2016; Cliff et al., 2017; Khacho and Slack, 2017a, b; Zhang et al., 2018; Jaiswal and Kimmel, 2019). Specific patterns of bioenergetic pathway activation influence cell fate decisions including the maintenance of self-renewal, induction of differentiation, homeostasis, activation state, and regenerative potential of distinct stem cell populations. It is now an established concept that cell identity itself, the outcome of multiple fate decisions during lineage development, is closely connected with particular metabolic states.

**FIGURE 1:**
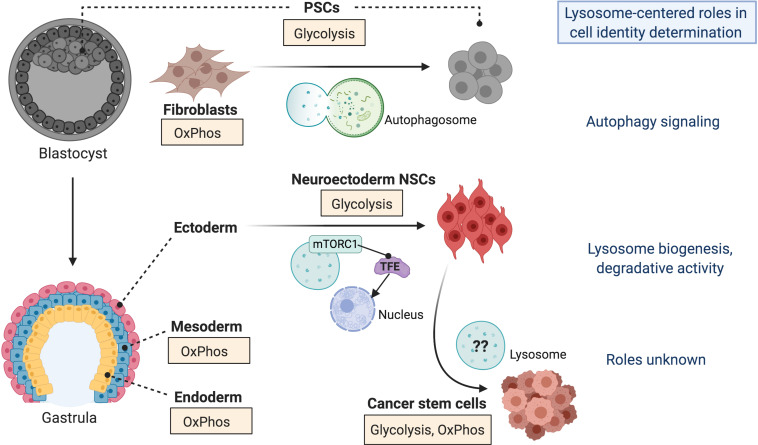
Lysosomes regulate stem cell identity transitions. Precise control of lysosome-autophagy signaling is required at an early stage during the reprogramming of somatic cells (i.e., fibroblasts) to induced pluripotent stem cells (PSCs) *in vitro*. *In vivo*, PSCs are located in the inner cell mass of the blastocyst during embryonic development, which gives rise to the gastrula from which the three primary germ layers (ectoderm, mesoderm, and endoderm) are born. Induction of ectodermal cells, specifically neural stem cells (NSCs) of the neuroectoderm, is accompanied by increased biogenesis and activity of lysosomes through mTORC1-regulated Micropthalmia/Transcription Factor E (TFE) nuclear organization. Alternatively, lysosome activation is associated with reduced endoderm and mesoderm differentiation. Given the prevalence of lysosome activity in various types of cancer, including those affecting the brain, it is possible that lysosomes drive the acquisition of cancer stem cell identity. The potential mechanisms are unknown. These stem cell identity transitions are also associated with metabolic reprogramming, whereby some cell types preferentially activate pathways requiring oxidative phosphorylation (OxPhos) whereas others rely on glycolysis. Cancer stem cells exhibit multiple strategies of metabolic reprogramming.

The connection between metabolic state and cell identity is well appreciated in the context of *in vitro* pluripotent stem cells (PSCs), including maintenance of their self-renewing state and the initial establishment of induced PSCs (iPSCs) from somatic cells such as fibroblasts. Transition to a glycolytic state from an oxidative one typical of somatic cells is universally required for both the acquisition and maintenance of pluripotency, critical for activation of self-renewal programs and inhibition of differentiation (reviewed in Zhang et al., 2018; Figure 1). There are subtleties to this rule however, as “naïve” PSCs, which functionally resemble the inner cell mass of the pre-implantation blastocyst (Figure 1), employ both OxPhos and glycolysis. This is in contrast to “primed” PSCs, a more developmentally mature population resembling cells within the post-implantation epiblast, which are predominantly glycolytic (Folmes et al., 2011; Zhang et al., 2018). This shift in metabolic preference between naïve and primed PSCs is accompanied by distinct changes in the expression profiles of developmental genes, which actively regulate glycolytic and OxPhos signaling patterns (Zhang et al., 2018). Subsequently, differentiation of PSCs into downstream lineages that parallel the mammalian germ layers is typically associated with transition from a glycolytic state to a preferential reliance on OxPhos (Figure 1). The metabolite a-ketoglutarate (aKG), generated through the mitochondrial OxPhos-dependent TCA cycle, functions in naïve PSCs to drive self-renewal but in primed PSCs to accelerate differentiation, a biphasic role attributed to the direct effect of aKG on stage-specific histone and DNA demethylation patterns and consequently epigenetic gene regulation (Carey et al., 2015; TeSlaa et al., 2016). Thus, a differential preference for OxPhos versus glycolysis signaling, reflecting unique needs for exogenous nutrients, regulatory co-factors, and oxygen and energy demands, parallels and can actively drive transitions between distinct stem cell identities.

The specification of ectodermal neural stem cells (NSCs) represents an exception to the paradigm that exit from pluripotency requires reprogramming to an OxPhos-dependent state (Figure 1), as a preference for glycolytic metabolism is maintained. Unlike in PSCs, however, glycolytic metabolism in the developing ectoderm appears to be driven by a reliance on glutamine as a primary metabolic substrate (Lees et al., 2018; Harvey et al., 2019; Vardhana et al., 2019). Furthermore, a proper balance of NSC self-renewal versus differentiation following their initial specification, as well as homeostasis of adult NSC populations, depends on a shift toward the oxidative fatty acid metabolic pathway. In mature populations this occurs specifically in quiescent NSCs, which are critical for the long-term maintenance of the stem cell pool and for neurogenic capacity in the brain, but not “activated” proliferative populations (Knobloch et al., 2017; Leeman et al., 2018; Bankaitis and Xie, 2019). Strikingly, inhibition of a single metabolite malonyl-CoA, a factor that drives activation of the fatty acid oxidation pathway, is sufficient to force quiescent NSCs into a proliferative state in a manner dependent on activation of the bone morphogenetic protein (BMP) signaling pathway (Knobloch et al., 2017). This again demonstrates a direct effect of metabolic state on cell fate regulation and underscores the integration of metabolic networks with developmental signaling pathways.

### Organelle Cooperation in Cell Metabolism

Metabolic pathways and the biological processes that fuel anabolic and catabolic activities are partitioned among distinct subcellular locations. Over the past decade, however, it has become increasingly clear that communication between organelles and cytoplasmic signaling pathways is critical for the dynamic regulation of bioenergetic networks that underlies cell and tissue homeostasis (reviewed in Schrader et al., 2015). The nucleus, mitochondria, endoplasmic reticulum (ER), and cellular vesicles (including lysosomes, autophagosomes, and peroxisomes) are historically defined, respectively, as regulators of gene expression, ATP production, protein and lipid production/export, and macromolecule degradation. Yet we now know that these organelles in fact exhibit multi-faceted roles within the cell, functionally and physically interacting with one another to cooperatively regulate processes that parallel or can directly impact metabolic control such as cell signaling, survival, immunity, and fate decisions.

Electron microscopy, immunofluorescence and time-lapse imaging analyses have revealed many organelles to be highly interconnected and dynamic structures (reviewed in Schrader et al., 2015; Lim and Zoncu, 2016). Regular fission and fusion events of mitochondria function to remodel their cristae and allow an exchange of intra-organelle and membrane components including ETC channel proteins, metabolites, calcium (Ca^2+^), and other ions. These interactions are essential for bioenergetic integrity, and consequently for the regulation of multiple facets of tissue development and maintenance (Khacho et al., 2016, 2017; Baker et al., 2019). Likewise, lysosomes move between the peri-nuclear region and the cell membrane in response to energy signaling demands through the mTORC1 signaling pathway (Starling et al., 2016; Hao et al., 2018), and they undergo regular fission and fusion events with one another (Ballabio and Bonifacino, 2020). This leads to changes in organelle size, vesicle tubulation and subsequent reformation of lysosomes from these hybrid organelles, presumably mediating transfer of membrane and lumenal contents among the broader lysosome population. Though lysosome dynamics appear to be important for bioenergetics and cell homeostasis, the mechanisms and direct consequences of these activities are not well understood (Saffi and Botelho, 2019; Ballabio and Bonifacino, 2020). Lysosomes have also long been known to fuse with autophagosome vesicles which carry excess and damaged cellular materials to the lysosome’s acidic lumen for degradation. Thus, lysosomes constitute the end point of the autophagy pathway, a ubiquitous cellular process critical for survival in the face of changing environments that impacts development, cell and tissue homeostasis, immunity, and disease predisposition (Chang, 2020).

In addition to “self-interactions,” multiple organelles in fact cooperate with one another in ways that can impact metabolic circuits. An emerging field has focused on the effects that by-products of mitochondrial metabolism can impart within the nucleus, including reactive oxygen species and multiple metabolites generated through the series of redox reactions that fuel oxidative metabolism. Many such metabolites, including αKG, NADH, and acetyl-coA, are produced through the TCA or in parallel pathways and subsequently function in the nucleus as co-factors for enzymes that regulate methylation and acetylation of DNA and histones. Mitochondrial metabolites can therefore significantly impact transcriptional regulation at an epigenetic level (Khacho et al., 2016; Harvey et al., 2019). This functional inter-organelle connection is a critical mechanism to control mitochondrial homeostasis and stability, as it permits activation of nuclear genes required for mitochondrial biogenesis. Disruption of mitochondria-nuclear connections can lead to DNA damage, calcium overload, developmental dysfunction, and disease including tumorigenesis (Guha and Avadhani, 2013; Cantó et al., 2015; Tu et al., 2018; Xia et al., 2019).

**FIGURE 2:**
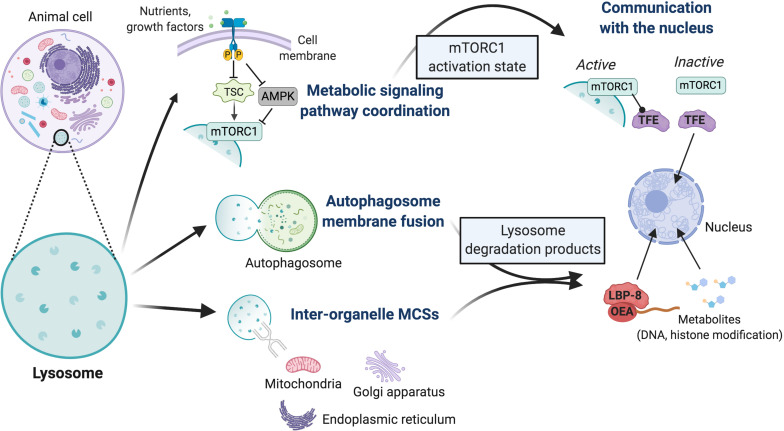
Overview of mechanisms by which lysosomes may impact cell fate decisions. Lysosomes interact physically with cell signaling pathways and other organelles; these interactions exhibit known or putative secondary impacts on transcriptional activity within the nucleus. The activation state of the mTORC1 and AMPK metabolic signaling pathways is coordinately regulated at the lysosome surface, with mTORC1 physically bound to the lysosome in its activated state. TSC is a potent mTORC1 inhibitor complex, whose inactivation underlies the multi-system tumor syndrome tuberous sclerosis. Lysosomes also mediate physical interactions with themselves or autophagosomes through membrane fusions, or with mitochondria, the endoplasmic reticulum, and Golgi apparatus through tethering proteins that establish inter-organelle membrane contact sites (MCSs). These contacts regulate fission-fusion events and inter-organelle transport of ions, lipids, and metabolites, which ultimately impacts the profile of lysosome degradation products. These direct physical interactions of the lysosome have secondary impacts on the nucleus. Currently, lysosomes are known to coordinate the nuclear activity of two classes of transcription factors. The subcellular localization of TFE family members, critical activators of lysosome biogenesis genes, is controlled by the activation state of mTORC1. In its active state, lysosome-bound mTORC1 physically retains TFE in the cytoplasm, while its dissociation from the lysosome upon inactivation leads to TFE release and nuclear translocation. Demonstrated in *C. elegans*, degradation products of the lysosomal lipase LIPL-4 form a lipid (OEA)-lipid chaperone protein (LBP-8) complex that translocates to the nucleus and promotes the transcriptional activity of nuclear receptor hormone receptors, factors that activate mitochondrial metabolism and oxidative stress response genes. Various metabolites are produced through lysosome-mediated digestion of excess or damaged organelles, proteins, and other cellular materials, and can be transported to the cytoplasm by membrane permeases. Given our insights into mitochondrial metabolites, these likely impact multiple biochemical pathways and nuclear activities through modification of DNA and histone proteins. Many of these mechanisms have been implicated in cell fate regulation; it largely remains to be determined which processes impact stem cell identity.

More recently, the lysosome has emerged as an organelle that also has direct functional connections to the nucleus. Dynamics of nutrient-sensing signaling complexes on the lysosome surface control the sub-cellular localization of the Micropthalmia/Transcription Factor E (MiT/TFE) family of transcription factors (herein referred to as ‘TFE’), critical regulators of genes that drive lysosome biogenesis and metabolic reprogramming (Settembre et al., 2011, 2012; Young et al., 2016; Figure 2). Additionally, the lipase LIPL-4 promotes longevity in *Caenorhabditis elegans* by harnessing its degradative activity within the lysosome to establish a lipid–protein chaperone complex that translocates to the nucleus and promotes the transcriptional activity of NHR-49 and NHR-80 nuclear hormone receptors (Folick et al., 2015). Nuclear hormone receptors regulate the expression of genes related to mitochondrial metabolism and the oxidative stress response, a mechanism linked to organismal longevity due to downstream activation of mitochondrial ß-oxidation and ETC activity (Folick et al., 2015; Ramachandran et al., 2019). Biochemical and high throughput metabolomic analyses revealed that the nuclear complex generated by LIPL-4 in *C. elegans* is comprised of the lipid species oleoylethanolamide (OEA) and the lipid chaperone LBP-8. All three elements of this signaling pathway are structurally or functionally conserved in mammals (LIPL-4 and NHR-80 are homologous to mammalian LIPA and HNF4, respectively; the mammalian lipid metabolism transcription factor PPARα is activated by OEA) (Fu et al., 2003; Folick et al., 2015; Ramachandran et al., 2019), thus similar lysosome-nuclear signaling mechanisms are likely to impact cell fate determinant transcriptional programs in mammalian systems.

Physical connections between organelles can take place through either direct membrane fusions or membrane contact sites (MCSs) mediated through tethering proteins. These connections permit the transport of metabolites and signaling molecules, lipid moieties, and ions like Ca^2+^ and iron that function as cofactors for biochemical reactions (Sheftel et al., 2007; Schrader et al., 2015; Todkar et al., 2017; Xia et al., 2019). They also participate in mediating and stabilizing structural processes, such as fission–fusion dynamics of mitochondria and lysosomes and the maturation of endosomes, including their trafficking along microtubules and ultimate fusion with lysosomes (Friedman et al., 2013). Membrane contact sites were in fact first observed, between the ER and mitochondria, by electron microscopy half a century ago (Copeland and Dalton, 1959). Despite this long history, we are only recently beginning to understand the molecular mechanisms that regulate these connections and the specificity of their biological effects.

Recent work demonstrated that in order to promote vascular modeling in the brain, reactive astrocytes develop clusters of mitochondrial–ER contacts to drive Ca^2+^ uptake from the ER. This was shown to be dependent on the mitochondrial fusion protein Mitofusin 2 (Mfn2), and thus is linked to dynamics in mitochondrial structure (Göbel et al., 2020). We now know that multiple organelles establish MCSs with other membrane-bound organelles or cell structures. This includes between the plasma membrane and the ER, which regulates Ca^2+^ dynamics between the extracellular space and the ER, the dominant Ca^2+^ storage site in the cell. Lysosomes have been observed to form MCSs with multiple organelles, including the ER, peroxisomes, Golgi apparatus, and mitochondria (Schrader et al., 2015; Todkar et al., 2017; Xia et al., 2019; Figure 2). A tethering complex that mediates mitochondrial–lysosome MCSs has been identified in yeast, where it is an important regulator of cell growth and is activated and maintained in response to metabolic activity (Hönscher et al., 2014). Alterations in the abundance of organelle MCSs are observed in cases of aberrant metabolic regulation and metabolic disorders, and gene knockout studies of proteins that comprise MCSs have begun to reveal that the integrity of at least some of these contacts is indeed crucial to proper metabolic regulation (Sebastián et al., 2012, 2016; Gordaliza-Alaguero et al., 2019).

### Organelle Inheritance and Cell Fate Determination

Gene and resulting protein expression patterns ultimately define and maintain a cell’s identity, and mitochondria are well-established regulators of gene expression programs. Alterations in mitochondrial content, fission and fusion dynamics, and signaling cascades including metabolite production and retrograde signaling to the nucleus has a profound impact on cell fate specification (Khacho et al., 2016; Khacho and Slack, 2017a). This encompasses broad populations including pluripotent, somatic and cancer stem cells (CSCs), from development through long-term homeostasis and aging. A compelling indicator that mitochondria play an instructive role in cell identity specification is that their segregation into the progeny of dividing stem cells has been observed to occur in a biased manner. For instance, during meiosis I of mammalian oocyte development mitochondria aggregate around the spindle pole, which will determine the plane of cell division. Upon division mitochondria are preferentially retained in the newly formed oocyte at the expense of the polar body, in a manner dependent on cell cycle progression and mitochondrial transport by the actin cytoskeleton (Dalton and Carroll, 2013). As the polar body is destined for degradation, this mechanism is thought to ensure the inheritance of mitochondria, and the consequent production of ATP and proper cell function, in cells destined to drive early embryo development.

Similarly, in budding yeast, mitochondria are partitioned in a biased manner at cell division such that the daughter bud cell receives a higher proportion of mitochondrial content at a cost to the mother cell (Böckler et al., 2017). As in mammalian oogenesis, this partitioning is dependent on fusion and transport of mitochondria on actin networks and is crucial for the maintenance of proper replicative lifespan of yeast bud cells. An elegant study that employed photoactivatable fluorescence tags as a relative measure of organelle age revealed that in actively expanding human mammary epithelial cultures, mitochondria exhibit biased distribution such that daughter stem cells with the highest potential for stemness receive predominantly young mitochondria (Katajisto et al., 2015). Genetic knock-down analyses targeting the structural fission proteins Parkin and Drp1 demonstrated that this regulated segregation also exploits fission–fusion dynamics.

As stem cells divide, molecular mechanisms converge to dictate whether their progeny will give rise to new-born stem cells through self-renewal, or instead to specialized differentiated cell types. One mechanism by which this is determined is regulation of the pattern in which cell fate determinant factors, including transcription factors, RNA molecules, signaling proteins, and organelles, are distributed among daughter cells (Venkei and Yamashita, 2018; Shlyakhtina et al., 2019). It is well established that stem cells divide with a spindle pole orientation that is either perpendicular to the surface of a defined stem cell niche or is at an altered angle relative to the niche. Though DNA is equally distributed between both daughter cells, the distribution of many cell fate determinants is polarized relative to the niche instead of the mitotic spindle pole. Therefore, perpendicular spindle orientations result in symmetric divisions in which both daughter cells receive a relatively equal complement of cytoplasmic material and consequently self-renew as clonal stem cells. Reduced angle divisions, however, are asymmetric, whereby cellular components are unevenly divided among daughter cells thus yielding one new stem cell and one differentiated progeny (Venkei and Yamashita, 2018).

The balance between symmetric self-renewing and asymmetric differentiative divisions is essential to tissue development and homeostasis, and misbalances can lead to diseases such as cytopenias or cancer. During early brain development, for example, a stem cell population expands first through symmetric divisions and subsequently through asymmetric divisions, which generate one daughter stem cell and one differentiated progeny (Julian et al., 2013; Matsuzaki and Shitamukai, 2015). In contrast, tissue homeostasis in the adult brain is maintained through a balance of symmetric self-renewing and asymmetric neurogenic divisions (Obernier et al., 2018). Ultimately, the pattern by which cell fate determinants are distributed among daughter cells is a critical factor directing cell identity.

In addition to mitochondria, other organelles exhibit biased segregation among progeny of dividing stem cells, suggesting that they may directly impact stem cell identity determination in their own right. Unequal inheritance of ER content has been observed in asymmetrically dividing stem cell populations in a number of model systems including *Drosophila melanogaster*, *Caenorhabditis elegans*, *Saccharomyces cerevisiae*, the ascidian embryo, and cultured human cells (Estrada et al., 2003; Poteryaev et al., 2005; Sardet et al., 2007; Smyth et al., 2015; Eritano et al., 2017). Asymmetric distribution of the ER has also recently been observed in symmetrically expanding epithelial stem cells very early stages of Drosophila development, through a mechanism dependent on directed positioning of the ER by the highly conserved ER membrane protein Jagunal (Eritano et al., 2017). Strikingly, this biased ER inheritance is observed immediately prior to the delamination event that precedes the transition to asymmetric neurogenic divisions and is restricted to a population of cells destined for neurogenesis. This strongly implies a direct role for the ER in stem cell fate determination; it remains to be determined, however, if asymmetric ER inheritance contributes actively to cell fate determination in mammalian systems.

Asymmetric inheritance of lysosomes and other cellular vesicles has also recently been causally linked to cell fate determination. Peroxisomes, vesicles carrying degradative enzymes involved in fatty acid and energy metabolism, have been observed in both yeast and human cells to exhibit asymmetric segregation during mitosis (Asare et al., 2017; Kumar et al., 2018). Using an unbiased RNA-sequencing and a directed RNA interference (RNAi) approach to profile epidermal stem cells and their newly differentiated progeny in developing mouse embryos, peroxisomes were shown to be critical for epidermal development through regulation of asymmetric divisions (Asare et al., 2017). These vesicles localize to spindle poles and are in fact necessary to control their alignment and the subsequent orientation of cell division relative to the stem cell niche. RNAi-mediated knockdown of the peroxisome membrane protein Pex11b completely disrupted epidermal development by altering cell cycle progression, symmetric and asymmetric division ratios and ultimately fate determination of the stem cell pool and the resulting tissue architecture.

Finally, in the first explicit demonstration that mammalian hematopoietic stem cells (HSCs) divide asymmetrically to control their fate, Loeffler et al. (2019) demonstrated that this asymmetry is paralleled by an uneven distribution of the cell’s degradative machinery, specifically lysosomes, autophagosomes, and mitophagososmes. Preferential inheritance of these vesicles into daughter cells destined for differentiation was a defining feature dictating the identity of HSC progeny, and was linked to the regulation of mitochondrial clearance, protein translation capacity, and signaling through the Notch–Numb pathway. Thus, compelling evidence from recent discoveries implicates multiple organelles, focused heavily on cellular metabolic vesicles such as lysosomes, as active regulators of stem cell divisions that dictate cell identity decisions.

## Lysosomes as Regulators of Stem Cell Identity

Lysosomes have long been viewed as the primary effectors of the cellular waste disposal system, functioning to degrade excess or damaged organelles and macromolecules by the greater than 60 hydrolytic enzymes present within their acidic lumen (Settembre et al., 2013; Perera and Zoncu, 2016). Lysosomes are formed by the fusion of secretory vesicles from the trans Golgi network with endocytic vesicles established by plasma membrane endocytosis. Lysosomes receive and digest intracellular components including excess proteins, lipids, carbohydrates, nucleic acids, and damaged organelles by autophagosomes, and thus represent a critical end point in the autophagy system (Chang, 2020; Figure 2). Lysosomes also digest extracellular macromolecules and materials such as aging cells, cell debris, bacteria, and viruses delivered through endocytosis and phagocytosis.

Lysosomal degradation reduces its substrates to fundamental building blocks of macromolecules including amino acids, sugars, lipids, and nucleic acids. These metabolites are either retained in the lysosome lumen to buffer their cytoplasmic concentrations, or are actively secreted into the cytoplasm by membrane permeases where they are reused by the cell to fuel biochemical and bioenergetic pathways, or exported out of the cell (Figure 2; Sagné et al., 2001; Rong et al., 2011; Jézégou et al., 2012; Liu et al., 2012; Verdon et al., 2017; Wyant et al., 2017). It is becoming increasingly clear that the processing and transport of lysosome-generated metabolites is highly regulated, and that mechanisms permitting contact with other organelles, such as MCS interaction with mitochondria, allow for bi-directional transport of metabolites and lipids that likely fuel metabolic and other biochemical pathways. Lysosomes also function as central players in coordinating supply and demand of critical molecules involved in bioenergetic signaling (Zhang et al., 2011b; Lim and Zoncu, 2016; Savini et al., 2019; Ballabio and Bonifacino, 2020). The signaling pathways and mechanisms that impact lysosome biogenesis and function are linked to many activities that implicate them as regulators of stem cell biology, including self-renewal and differentiation decisions, cell death, and organismal aging and longevity (Ramachandran et al., 2019; Savini et al., 2019; Ballabio and Bonifacino, 2020). An important role for lysosomes in cell identity determination is emerging, yet the mechanisms by which they may drive metabolic reprogramming and impact cell fate specification, and if these activities are indeed linked, are unclear.

### Metabolic Signaling Pathways Converge at the Lysosome

Metabolic pathways and the cell biological processes with which they integrate are dynamic. Given that cells are regularly exposed to changing environmental conditions, mechanisms to finely regulate the balance between anabolic and catabolic activities are critical for cell viability, proper function, and homeostasis. This coordination is achieved by hard-wired signaling mechanisms that sense the level of available nutrients and other environmental signals like oxygen, energy status, stress markers, and growth factors, and accordingly relay downstream signals through phosphorylation events. The mammalian target of rapamycin complex 1 (mTORC1) and AMP-activated kinase (AMPK) are the primary mammalian nutrient-sensing kinases, and they function in opposition to one another to control the catabolic–anabolic balance. A major breakthrough in our understanding of metabolic coordination, and one that underscored a profound physical connection between metabolic signaling and the lysosome, is that the activity status of these kinases is in fact regulated on the lysosome surface (Sancak et al., 2010; Zhang et al., 2014; Savini et al., 2019). In the decade since this discovery, we now understand that mTORC1 is in fact recruited to and activated at the lysosome in a two-step process, requiring both sufficient nutrient and growth factor signaling. Nutrient signaling, translated through amino acid availability, activates heterodimeric Rag GTPases which physically recruit mTORC1 to the lysosome (Yang et al., 2017; Lawrence et al., 2018). Subsequently, mTORC1 interacts with the small GTPase Rheb already located on the lysosome surface. If growth factor signaling permits, the GTPase-activating protein “tuberous sclerosis complex” (TSC) will be inhibited, thus promoting activation of Rheb and consequently of mTORC1 (Tee et al., 2003; Menon et al., 2014; Savini et al., 2019).

Hyper-active mTORC1 functions at the lysosome to phosphorylate downstream targets that promote anabolic cellular processes including protein translation, ribosome and lipid biogenesis, cell growth, and mitochondrial metabolism (Delaney et al., 2014; Lawrence and Zoncu, 2019; Savini et al., 2019; Ballabio and Bonifacino, 2020). Conversely, mTORC1 inhibits catabolic activities, predominantly lysosome biogenesis, and autophagy signaling. The primary catabolic targets include the autophagosome assembly protein ULK-1 and members of the TFE transcription factor family, key factors in the activation of lysosome biogenesis genes (Sardiello et al., 2009; Palmieri et al., 2011; Settembre et al., 2011; Martina et al., 2014; Annunziata et al., 2019; Savini et al., 2019).

AMP-activated kinase is activated in response to low nutrient levels, when intracellular ATP levels are low and AMP is high, and functions to promote catabolism and inhibit anabolism largely by countering mTORC1 activities (Figure 2). mTORC1 and AMPK are regulated reciprocally by the lysosome-bound v-ATPase that permits nutrient sensing of amino acid levels inside the lysosome in addition to cytoplasmic sensing (Zhang et al., 2014). Likewise, the cytoplasmic/lysosomal and nuclear localization of TFE transcription factors is controlled in opposing ways by mTORC1 and AMPK (Martina et al., 2012, 2014; Roczniak-Ferguson et al., 2012; Settembre et al., 2012; Young et al., 2016; Saxton and Sabatini, 2017; Napolitano et al., 2018; Asrani et al., 2019). Corroborating a coordinated mechanism that impacts the dynamics of lysosome biogenesis as well as cell fate determination, it was recently shown in the amoeba Dictyostelium that activated mTORC1 and AMPK reciprocally regulate an overlapping set of genes that dictate a fate switch between progenitor cell growth and differentiation (Jaiswal and Kimmel, 2019).

Metabolic signaling pathways centered at the lysosome are in fact important regulators of cell fate transitions. The ability to balance mTORC1 activity is critical for proper stem cell fate decisions between self-renewal and differentiation in development and aging in a number of somatic cell types, with mTORC1 hyper-activation typically driving aberrant lineage differentiation (Delaney et al., 2014; Meng et al., 2018). Loss of the TSC complex members hamartin (TSC1) or tuberin (TSC2), which drives constitutive mTORC1 activation, leads to the development of a multi-system low-grade tumor and neoplastic disorder called tuberous sclerosis. In this condition, lesions are characterized by enlarged cells expressing an atypical mixture of lineage-specific stem cell and immature differentiation markers (Delaney et al., 2014).

We and others have additionally found that the capacity to repress the mTORC1 pathway is essential for the reprogramming of both mouse and human somatic cells to iPSCs (He et al., 2012; Wang et al., 2013; Wu et al., 2015; Armstrong et al., 2017; Julian et al., 2017a). This requirement has been linked to both p53-dependent cell death regulation and an intriguing mechanism whereby the critical cell fate determinant SOX2 transcriptionally suppresses mTORC1, at a very early stage of iPSC reprogramming, to permit transient activation of autophagy at this critical time-point of cell fate specification (Wang et al., 2013; Armstrong et al., 2017). mTORC1 inhibition by the core pluripotency factors during early reprogramming is also required to permit the reduction in mitochondrial mass (Wang et al., 2017, 2013) that coincides with the lower OxPhos activity typical of PSCs (Folmes et al., 2011). Demonstrating that lysosomes and other organelles can affect cell identity determination in distinct ways, this mitochondrial remodeling was strikingly independent of autophagy, evidenced by findings that mitochondria-lysosome co-localization could not be observed and lysosom inhibition with bafilomycin or shRNA targeted knock-down of autophagy regulators (Atg5, Beclin or Vps34) did not affect mitochondrial mass (Wang et al., 2013). The requirement for mTORC1 inhibition in regulating pluripotency appears to be limited to the initial stage of cell identity determination, as we and others have demonstrated that the loss or reduction of TSC2, limiting the capacity to inhibit mTORC1, does not affect the maintenance of a pluripotent state in established human PSCs (Julian et al., 2017a; Blair et al., 2018; Delaney et al., 2019). Thus, mTORC1 inhibition and consequent autophagy activation appears to be critical specifically at a very early stage of cell fate transition (Figure 1).

### Emerging Evidence for Lysosomes in Lineage-Specific Fate Determination

Though lysosome biogenesis and activity have been linked to stem cell fate decisions from development through aging, it has been unclear if lysosomes are drivers of these events or instead, passengers in the process. The acquisition and maintenance of pluripotency, for instance, requires metabolic reprogramming to a highly glycolytic state (Zhu et al., 2010; Folmes et al., 2011; Zhang et al., 2011a; Hansson et al., 2012; Panopoulos et al., 2012; Wang et al., 2013; Prigione et al., 2014) and *in vitro* differentiation of PSCs to downstream lineages correlates with further metabolic changes (Chung et al., 2007; TeSlaa et al., 2016; Betschinger, 2017; Cliff et al., 2017; Lees et al., 2018; Zhang et al., 2018). Our current understanding of the metabolic mechanisms driving these fate transitions is focused on the mitochondria with limited insight regarding the potential impact of other organelles. Landmark studies over the past few years have provided strong evidence that lysosomes can indeed function as drivers of both metabolic network reprogramming and stem cell fate decisions that affect cell identity determination.

It is now clear that lysosomes function as active drivers of PSC differentiation through their physical and functional interactions with the nutrient-sensing mTORC1, Rag GTPase, and AMPK complexes (Young et al., 2016; Villegas et al., 2019; Figure 2). Villegas et al. (2019) performed a genome-wide CRISPR/Cas9 screen in mouse embryonic stem cells (ESCs) under culture conditions that promoted either self-renewal or differentiation, to identify factors whose loss would impede exit from the pluripotent state. This unbiased approach identified a number of factors related to lysosome biogenesis and function as critical regulators of PSC differentiation (Villegas et al., 2019). These include genes that ensure lysosome degradative activity, as well as the Rag proteins and components of the Ragulator complex that recruits Rag proteins to the lysosome surface, necessary for control of mTORC1 activity. Also identified as a critical regulator was the tumor suppressor Folliculin, which normally translates amino acid levels to Ragulator/Rag permitting non-nuclear recruitment and sequestration of Tfe3. The authors discovered that loss of these factors leads to constitutive localization of Tfe3 in the nucleus, which is known to directly drive gene expression programs related to lysosome biogenesis and metabolic signaling. This was confirmed in the study using RNA-sequencing, a strategy that revealed a secondary transcriptional response of Tfe3 that enforces a sustained pluripotency program and represses transcriptional programs associated with peri-implantation development and neural lineage differentiation.

Similarly, mouse ESCs lacking functional AMPK, which antagonizes mTORC1 activation at the lysosome and thereby inhibits its phosphorylation-induced retention of TFE proteins in the cytoplasm, exhibit profound differentiation defects. *AMPK*^–/–^ ESCs maintain pluripotency but fail to generate chimeric embryos and exhibit a preference for ectodermal compared to endodermal differentiation, due to hypo-phosphorylation of Tfeb and its reduced nuclear localization (Young et al., 2016). This study and others position coordinated regulation of AMPK and mTORC1 signaling to be an essential regulatory node in PSCs that determines an ectodermal or endodermal cell fate, with hyper-active mTORC1 signaling consistently leading to preferential ectodermal differentiation (Zhou et al., 2009; Young et al., 2016; Delaney et al., 2019; Jaiswal and Kimmel, 2019).

Underscoring an essential role for lysosomes in cell fate decisions particularly in NSC pools, differences in proteasome and lysosome-autophagy activity have recently been revealed as defining factors between proliferative “activated” NSC populations versus aged quiescent NSCs, whose accumulation is strongly associated with age-related cognitive decline (Leeman et al., 2018). The authors employed RNA-sequencing, again an unbiased approach, to profile transcriptional signatures of activated and quiescent cell populations within the NSC pool isolated from young and aged mice. Differences in metabolic profiles and active components of the proteostasis system, specifically the proteasome and lysosome machinery, were the most profound signatures discerning proliferative from quiescent NSCs. Transcriptional signatures and functional experiments revealed that the proteostasis machinery is highly functional in activated NSCs, whereas activity is low in quiescent cells. As quiescent NSCs age, their lysosomes become significantly enlarged and accumulate increasing amounts of insoluble aggregated proteins, which is due to a lack of efficient clearance by the lysosome. Activation of lysosome activity by TFEB expression in quiescent NSCs permitted clearance of protein aggregates and, strikingly, altered the fate of these cells to an activated state.

Our recent work further implicates lysosome activation as a critical requirement for NSC fate regulation, in the initial determination of neural lineage identity (Delaney et al., 2019; Figure 1). We developed a modeling system of the multi-system tumor disorder tuberous sclerosis by engineering inactivating *TSC2* mutations in human PSCs, thereby inducing mTORC1 hyperactivation, and subsequently differentiating them in a directed manner into NSCs and the developmentally related neural crest (NC). These two lineages are the presumed derivative cell types of the diverse lesions and tumors in tuberous sclerosis patients (Delaney et al., 2014). Seeking to determine the mechanisms by which normal (WT) and *TSC2*^–/–^ PSCs commit to an NSC or NC fate, we employed RNA-sequencing and functional analysis approaches to identify the primary biological processes at play during the transition from pluripotency to the new cellular identity. An increase in lysosome content, driven by an acute activation of a proteostasis stress response, was evident at very early stages of PSC–NSC induction, but not during NC specification. This was observed in WT cells and was further exacerbated in cells lacking TSC2. Of note, a recent study revealed that cells undergoing NSC specification undergo a reprogramming of protein chaperone networks, which are intimately connected with proteostatic mechanisms; this finding underscores the importance of proteostasis signaling integrity during early NSC development (Vonk et al., 2020). Furthermore, lysosome content changed dynamically throughout NSC induction in *TSC2*^–/–^ cells, and these patterns correlated with alterations in the expression of cell fate markers (Delaney et al., 2019).

The aberrant lysosome activation driven by TSC2-deficiency was transient during neural development but became reactivated with time as NSC cultures were aged, in a manner that paralleled altered expression of cell fate markers (Delaney et al., 2019). These findings suggest a close association of lysosome biogenesis with cell fate in NSC populations, both during their initial specification and long-term maintenance. Our findings that early induced NSCs lacking TSC2 activate lysosome biogenesis, while maintaining degradative activity through autophagy, in parallel with hyperactive mTORC1 signaling reveals the existence of early adaptive mechanisms in metabolic circuits to ensure proper signaling for lineage development. A recent study suggests that autophagy-dependent lipid metabolism is an important aspect of these adaptations (Wang et al., 2019). We also found that increased lysosome biogenesis preceded later changes in mitochondrial content, which correlated with aberrant activation of oxidative metabolism uniquely in *TSC2*^–/–^ NSCs (Delaney et al., 2019). This demonstrates that lysosome activation can be a driver of metabolic and cell fate changes, at least in the neural lineage, and is not simply a downstream consequence of mitochondrial alterations (Figure 1).

### Implications for Disease Mechanisms and Treatment

Many human disorders, ranging from developmental to aging-induced conditions and various forms of cancer, exhibit aberrant lysosome activity as a known or predicted factor contributing to pathogenic phenotypes. The over 50 rare genetic metabolic conditions under the umbrella of “lysosomal storage diseases” typically present early in life, yet we now know that they share many clinical, pathological and genetic features with age-related neurological conditions, including frontotemporal dementia, Alzheimer’s and Parkinson’s disease (Fraldi et al., 2016). Shared pathological phenotypes are typified by metabolic dysfunction and an accumulation of aggregated proteins as a consequence of insufficient proteostasis mechanisms that include altered lysosome activity. Retrospective studies and PSC-based disease modeling approaches, which permit detailed analysis of the biological effects of allele variants starting from the earliest stages of cell lineage development, have begun to reveal that gene mutations associated with adult onset degenerative conditions can confer measurable phenotypes during development or at much earlier stages than previously considered (Delaney et al., 2019; Smits et al., 2019; Zhu et al., 2019; Johnson et al., 2020). Examples include altered stem cell proliferation, differentiation decisions, metabolic and proteostasis signaling in studies of tuberous sclerosis, Alzheimer’s and Parkinson’s disease (Delaney et al., 2019; Smits et al., 2019; Zhu et al., 2019). Lysosomal dysfunction and metabolic reprogramming have also been implicated in neurological conditions not directly associated with degeneration, including psychiatric and seizure disorders; likewise, many lysosomal storage diseases and other conditions, such as inherited tumor syndromes, present with psychiatric disease and often neurodegeneration (Staretz-Chacham et al., 2010; Marques and Saftig, 2019).

It is therefore clear that the brain, and the neural cell lineage that builds this organ, are highly dependent on the lysosome for proteostasis regulation and is particularly sensitive to developing disease when faced with lysosome dysfunction. This neural lineage proteostasis sensitivity is often attributed to the fact that neurons are highly active biologically but unable to divide and thereby have no progeny through which to dilute protein aggregates and aberrant regulatory machinery. Our recent work and that of others discussed in this review (Zhou et al., 2009; Young et al., 2016; Leeman et al., 2018; Delaney et al., 2019; Jaiswal and Kimmel, 2019) suggest that unique proteostatic mechanisms with a particular dependency on lysosome-mediated degradation are in fact active at the earliest stages of neural lineage development. Moreover, these findings imply that lysosome activation is critical for NSC fate determination, suggesting a hard-wired integration of lysosomal signaling mechanisms with metabolic and transcriptional programs that coordinate NSC identity.

Hard-wiring of lysosomal signaling in NSCs has implications for understanding susceptibility to neurological disease, as well as the stage-specific drivers of diseases that are associated with changes in markers of cell fate and identity, such as cancer (Figure 1). Similar to stem cells transitioning between cell fates, tumorigenesis is associated with dramatic metabolic alterations. These are typically unique to each tumor type but are common conceptually, given that significant metabolic remodeling occurs at each phase from pre-malignant lesion, to aggressive primary tumor, and consequently metastasis (Faubert et al., 2020). These changes occur as tumors, typically thought to be driven by populations of adaptive CSCs, face ever changing environments that are commonly low in oxygen and standard energy sources (Nazio et al., 2019).

Many new and developing therapeutic strategies for cancer are directed toward targeting metabolic vulnerabilities of the tumor. Inhibition of lysosome activity by targeting autophagy signaling, using lysosomotropic agents like bafilomycin or chloroquine derivatives, or degradative capacity and ion transport are popular among these approaches (Nazio et al., 2019; Méndez-Lucas et al., 2020). Yet, despite the clear vulnerability of metabolic and lysosome-based networks in cancer, current lysosome-targeted drugs have had limited success in clinical trials, often failing to provide adequate tumor-specific toxicity at least as single agents. We posit that a deeper understanding of the organelle-based processes that drive CSC adaptations is one avenue that will lead to more targeted selection of therapeutic agents based on the tumorigenic stage, the lineage identity of resident CSCs, and the driving biological mechanisms (Figure 1).

Efforts aimed at elucidating the extent to which lysosome dynamics influence cell fate specification in stem cell populations are likely to yield important therapeutic insights. Using CSCs as an example, an important approach is to discern whether altered lysosome signaling in a given tumor type is an early precipitating event in cell fate and metabolic dysfunction, as we observed in our human stem cell model of tuberous sclerosis (Delaney et al., 2019), or instead a secondary event, potentially consequent to mitochondrial reprogramming. An improved understanding of the driving and secondary adaptive mechanisms that contribute to stem cell-based disease has high potential to reveal effective stage-specific biomarkers and to guide rational development of therapeutic strategies.

Lysosomes are complex organelles, with an ever-growing list of associated biological functions with the potential to impact stem cell fate and identity determination (Figure 2). Beyond their canonical role of degrading macromolecules through their acidic environment and portfolio of hydrolytic enzymes, they also act as nutrient sensors, platforms for signaling pathways, regulators of lipid production and transport, and mediators of ion, nucleic acid, amino acid, and metabolite signaling and transport. Lysosomes are also structurally dynamic, undergoing fission and fusion events, relying on their connection to the actin cytoskeleton, and can physically and functionally interact with multiple organelles. Additionally, they have emerged as regulators of transcriptional programs, for instance by modulating TFE sub-cellular localization and engaging lipid metabolites for nuclear signaling.

It is likely that many more functions remain to be uncovered by which the lysosome impacts stem cell fate. For instance, mTORC1 and AMPK are not the only signaling hubs located at the lysosome surface; additionally, there are likely many more mechanisms to be uncovered that contribute to mTORC1-dependent molecular sensing (Young et al., 2016; Savini et al., 2019; Ballabio and Bonifacino, 2020). Moving forward it will be critical to understand the mechanisms by which lysosomes impact cell fate specification, especially their impacts on transcriptional and translational programs. For example, are there additional mechanisms outside of lipid and TFE signaling by which the lysosome affects transcriptional programs? Possibilities include direct regulation of the expression or localization of additional transcription factors, or the production and transport of metabolites that affect epigenetic signatures. Lysosome metabolite profiling represents a particularly exciting and newly developing area of research that will undoubtedly help deepen our understanding of the mechanisms by which this organelle impacts stem cell identity determination, and furthermore how it may be efficiently targeted in cancer and disease states (Abu-Remaileh et al., 2017). Acquiring a deeper understanding of lysosome-based signaling at the metabolite level and beyond has the potential to discover targeted biomarkers and therapeutic approaches for stem cell-based disease, and to improve strategies for tissue regeneration.

## Concluding Remarks

Regulation of stem cell fate decisions is heavily predicated on the integration of transcriptional and metabolic signaling networks. Our understanding of the metabolic impacts on cell fate have been largely limited to the mitochondria, yet it is becoming increasingly evident that multiple organelles interact with one another in diverse ways to regulate cell fate. The lysosome is now well appreciated as not only the waste disposal center of the cell but as an active and multifunctional player in coordinating metabolic networks; exciting work over the past few years has provided compelling evidence that lysosome-based activities are in fact crucial for regulating cell identity determination and other fate decisions. The lysosome is a multi-functional organelle whose activities and regulatory partners are still being uncovered. Thus, focusing efforts on unraveling the biological impacts of lysosome signaling on cell fate regulation has high potential to significantly impact our understanding of tissue development and aging, disease predisposition and pathogenesis, as well as disease treatment and regenerative medicine strategies.

## Author Contributions

LJ wrote the manuscript and prepared the figures. WS reviewed and edited the manuscript, provided intellectual insights, and acquired funds for publication. Both authors contributed to the article and approved the submitted version.

## Conflict of Interest

The authors declare that the research was conducted in the absence of any commercial or financial relationships that could be construed as a potential conflict of interest.
